# Supplementary material for: Non-response in a national health survey in Germany: An intersectionality-informed multilevel analysis of individual heterogeneity and discriminatory accuracy
Source: PLoS One. 2020 Aug 10;15(8):e0237349. doi: 10.1371/journal.pone.0237349 (PMC7416954; doi:10.1371/journal.pone.0237349)
Supplement: S1 Table — Intersectional strata are ranked by predicted proportions of non-responders. (DOCX) [file pone.0237349.s001.docx]

**S1 Table**

| Stratum rank | Age | | | Sex/gender | | Marital status | | Educational level | | Number of observations | Number of non-responders | Predicted proportion of non-responders | | | Intersectional effects | | |
| --- | --- | --- | --- | --- | --- | --- | --- | --- | --- | --- | --- | --- | --- | --- | --- | --- | --- |
|  | 18-39 | 40-59 | 60-79 | male | fem. | marr. | not marr. | high | low | N | n |  | 95% CI | |  | 95% CI | |
| 1 |  | X |  |  | X | X |  | X |  | 546 | 147 | 22,85 | 19,26 | 26,57 | 0,04 | -4,69 | 4,60 |
| 2 |  |  | X | X |  | X |  | X |  | 390 | 115 | 26,58 | 21,99 | 31,24 | -1,80 | -7,89 | 3,34 |
| 3 |  | X |  |  | X |  | X | X |  | 176 | 51 | 27,02 | 20,87 | 33,32 | -2,14 | -7,96 | 3,45 |
| 4 | X |  |  | X |  | X |  | X |  | 213 | 65 | 27,34 | 21,98 | 33,02 | -0,29 | -5,93 | 5,55 |
| 5 |  | X |  | X |  | X |  | X |  | 470 | 155 | 27,41 | 23,03 | 32,47 | 3,10 | -1,73 | 9,12 |
| 6 |  |  | X |  | X | X |  | X |  | 264 | 84 | 27,89 | 23,18 | 33,25 | 1,17 | -4,07 | 6,86 |
| 7 | X |  |  |  | X | X |  | X |  | 351 | 123 | 29,50 | 24,14 | 34,99 | 3,50 | -1,43 | 9,67 |
| 8 | X |  |  |  | X |  | X | X |  | 752 | 245 | 29,99 | 26,23 | 33,74 | -2,86 | -9,19 | 2,21 |
| 9 | X |  |  | X |  |  | X | X |  | 762 | 261 | 31,69 | 27,87 | 35,80 | -3,02 | -9,13 | 2,02 |
| 10 |  |  | X |  | X |  | X | X |  | 173 | 62 | 33,14 | 26,59 | 39,82 | -0,55 | -7,01 | 5,37 |
| 11 |  | X |  | X |  |  | X | X |  | 189 | 75 | 33,50 | 27,44 | 41,04 | 2,59 | -2,98 | 9,61 |
| 12 |  | X |  | X |  | X |  |  | X | 209 | 76 | 34,75 | 28,19 | 40,82 | -1,37 | -7,78 | 4,79 |
| 13 |  | X |  |  | X | X |  |  | X | 182 | 72 | 35,37 | 28,75 | 42,46 | 1,15 | -5,31 | 8,10 |
| 14 |  |  | X | X |  |  | X | X |  | 77 | 31 | 35,66 | 27,74 | 44,94 | 0,08 | -7,55 | 7,29 |
| 15 | X |  |  |  | X | X |  |  | X | 81 | 29 | 36,66 | 27,76 | 45,42 | -1,55 | -9,28 | 5,99 |
| 16 |  |  | X |  | X | X |  |  | X | 354 | 133 | 37,02 | 31,41 | 42,84 | -2,07 | -8,95 | 3,86 |
| 17 | X |  |  | X |  | X |  |  | X | 72 | 29 | 38,84 | 29,92 | 47,31 | -1,36 | -8,50 | 5,93 |
| 18 |  | X |  |  | X |  | X |  | X | 66 | 23 | 39,12 | 29,57 | 48,07 | -2,89 | -12,16 | 3,94 |
| 19 |  |  | X | X |  | X |  |  | X | 411 | 171 | 39,53 | 34,13 | 44,85 | -1,55 | -7,92 | 4,58 |
| 20 |  | X |  | X |  |  | X |  | X | 76 | 32 | 42,56 | 33,18 | 51,83 | -1,50 | -9,27 | 5,64 |
| 21 | X |  |  |  | X |  | X |  | X | 120 | 62 | 47,70 | 40,24 | 55,67 | 1,42 | -5,92 | 9,05 |
| 22 |  |  | X | X |  |  | X |  | X | 85 | 42 | 49,86 | 41,60 | 58,63 | 0,59 | -6,90 | 8,70 |
| 23 |  |  | X |  | X |  | X |  | X | 246 | 140 | 52,02 | 45,11 | 59,41 | 4,83 | -2,04 | 13,06 |
| 24 | X |  |  | X |  |  | X |  | X | 167 | 97 | 52,93 | 44,80 | 61,22 | 4,59 | -2,34 | 13,65 |
| fem.: female, marr.: married, not marr.: not married, CI: credible interval | | | | | | | | | | | | | | | | | |
