# Supplementary material for: Non-response in a national health survey in Germany: An intersectionality-informed multilevel analysis of individual heterogeneity and discriminatory accuracy
Source: PLoS One. 2020 Aug 10;15(8):e0237349. doi: 10.1371/journal.pone.0237349 (PMC7416954; doi:10.1371/journal.pone.0237349)
Supplement: S2 Table — Intersectional strata are ranked by predicted proportions of non-responders. (DOCX) [file pone.0237349.s002.docx]

**S2 Table**

| Stratum rank | Age | | | Sex/gender | | Marital status | | Educational level | | Subjecitve health | | Number of observations | Number of non-responders | Predicted proportion of non-responders | | | Intersectional effects | | |
| --- | --- | --- | --- | --- | --- | --- | --- | --- | --- | --- | --- | --- | --- | --- | --- | --- | --- | --- | --- |
|  | 18-39 | 40-59 | 60-79 | male | fem. | marr. | not marr. | high | low | good | bad* | N | n |  | 95% CI | |  | 95% CI | |
| 1 |  | X |  |  | X | X |  | X |  | X |  | 433 | 105 | 20,64 | 17,02 | 24,87 | -0,85 | -5,07 | 3,58 |
| 2 |  |  | X | X |  | X |  | X |  | X |  | 248 | 66 | 23,55 | 18,36 | 28,79 | -1,73 | -7,25 | 3,41 |
| 3 |  |  | X |  | X | X |  | X |  | X |  | 172 | 51 | 24,73 | 19,35 | 30,82 | 1,00 | -3,98 | 6,85 |
| 4 |  | X |  |  | X |  | X | X |  | X |  | 132 | 37 | 25,91 | 19,81 | 32,47 | -1,40 | -7,47 | 4,86 |
| 5 | X |  |  | X |  | X |  | X |  | X |  | 198 | 58 | 25,92 | 20,78 | 31,47 | -0,66 | -5,97 | 5,21 |
| 6 |  | X |  | X |  | X |  | X |  | X |  | 367 | 121 | 26,79 | 22,12 | 32,12 | 3,86 | -0,82 | 9,42 |
| 7 |  | X |  | X |  | X |  | X |  |  | X | 103 | 34 | 26,79 | 22,12 | 32,12 | -0,57 | -6,85 | 6,62 |
| 8 |  |  | X |  | X |  | X | X |  | X |  | 103 | 28 | 27,51 | 20,70 | 34,71 | -2,41 | -9,76 | 4,08 |
| 9 | X |  |  |  | X |  | X | X |  | X |  | 669 | 215 | 29,20 | 25,20 | 32,96 | -2,13 | -7,40 | 2,46 |
| 10 | X |  |  |  | X | X |  | X |  | X |  | 319 | 113 | 29,29 | 24,00 | 34,83 | 4,32 | -0,79 | 10,24 |
| 11 |  | X |  | X |  |  | X | X |  | X |  | 142 | 50 | 29,90 | 23,69 | 37,22 | 0,89 | -5,39 | 6,93 |
| 12 |  |  | X |  | X | X |  |  | X | X |  | 165 | 49 | 30,33 | 23,79 | 37,15 | -3,63 | -10,74 | 2,53 |
| 13 |  | X |  |  | X | X |  | X |  |  | X | 113 | 42 | 30,89 | 24,15 | 38,53 | 1,93 | -3,86 | 9,22 |
| 14 | X |  |  | X |  |  | X | X |  | X |  | 700 | 239 | 31,13 | 27,02 | 35,18 | -2,04 | -7,86 | 2,82 |
| 15 |  | X |  | X |  | X |  |  | X | X |  | 149 | 49 | 31,34 | 24,63 | 37,59 | -1,65 | -8,38 | 4,72 |
| 16 |  |  | X |  | X | X |  | X |  |  | X | 92 | 33 | 32,30 | 25,46 | 40,16 | 0,65 | -5,37 | 7,85 |
| 17 |  |  | X | X |  | X |  |  | X | X |  | 207 | 70 | 32,46 | 25,92 | 39,25 | -3,39 | -10,68 | 2,73 |
| 18 |  |  | X | X |  |  | X | X |  | X |  | 42 | 16 | 32,46 | 23,80 | 42,11 | 0,75 | -6,59 | 8,51 |
| 19 |  |  | X | X |  | X |  | X |  |  | X | 142 | 49 | 32,57 | 25,92 | 39,47 | -0,92 | -8,04 | 5,55 |
| 20 | X |  |  |  | X | X |  | X |  |  | X | 32 | 10 | 32,75 | 23,82 | 42,37 | -0,39 | -8,10 | 8,10 |
| 21 |  | X |  |  | X | X |  |  | X | X |  | 116 | 46 | 33,15 | 26,51 | 41,57 | 1,98 | -4,33 | 9,63 |
| 22 |  | X |  |  | X |  | X | X |  |  | X | 44 | 14 | 34,07 | 24,51 | 43,01 | -1,79 | -9,67 | 5,26 |
| 23 | X |  |  |  | X | X |  |  | X | X |  | 62 | 22 | 35,04 | 26,80 | 43,86 | -0,47 | -7,98 | 7,05 |
| 24 | X |  |  | X |  | X |  | X |  |  | X | 15 | 7 | 35,86 | 25,96 | 46,60 | 0,84 | -7,00 | 10,63 |
| fem.: female, marr.: married, not marr.: not married, *: moderate or bad, CI: credible interval | | | | | | | | | | | | | | | | | | | |

S2 Table (continued)

| Stratum rank | Age | | | Sex/gender | | Marital status | | Educational level | | Subjecitve health | | Number of observations | Number of non-responders | predicted proportion of non-responders | | | intersectional effects | | |
| --- | --- | --- | --- | --- | --- | --- | --- | --- | --- | --- | --- | --- | --- | --- | --- | --- | --- | --- | --- |
|  | 18-39 | 40-59 | 60-79 | male | fem. | marr. | not marr. | high | low | good | bad* | N | n |  | 95% CI | |  | 95% CI | |
| 25 | X |  |  | X |  | X |  |  | X | X |  | 62 | 25 | 36,99 | 28,15 | 45,90 | -0,44 | -7,83 | 6,93 |
| 26 |  | X |  |  | X |  | X |  | X | X |  | 43 | 17 | 37,79 | 28,48 | 48,02 | -0,53 | -9,34 | 7,90 |
| 27 | X |  |  |  | X |  | X | X |  |  | X | 83 | 30 | 38,08 | 29,43 | 46,38 | -2,38 | -9,75 | 4,65 |
| 28 |  | X |  |  | X | X |  |  | X |  | X | 66 | 26 | 39,44 | 30,21 | 47,97 | -0,81 | -8,74 | 6,75 |
| 29 | X |  |  | X |  |  | X | X |  |  | X | 62 | 22 | 39,79 | 30,76 | 48,73 | -2,70 | -11,44 | 5,65 |
| 30 |  |  | X | X |  |  | X | X |  |  | X | 35 | 15 | 40,22 | 30,62 | 50,82 | -0,62 | -9,32 | 7,44 |
| 31 |  | X |  | X |  |  | X |  | X | X |  | 45 | 20 | 40,64 | 31,42 | 51,22 | 0,33 | -7,99 | 9,44 |
| 32 |  | X |  | X |  |  | X | X |  |  | X | 47 | 25 | 41,38 | 32,12 | 52,37 | 3,57 | -4,39 | 13,41 |
| 33 |  |  | X |  | X |  | X | X |  |  | X | 70 | 34 | 41,46 | 33,12 | 50,96 | 2,61 | 5,07 | 11,44 |
| 34 |  |  | X |  | X |  | X |  | X | X |  | 97 | 45 | 41,85 | 34,40 | 50,71 | 0,49 | -6,49 | 8,69 |
| 35 |  | X |  | X |  | X |  |  | X |  | X | 60 | 27 | 42,82 | 34,02 | 52,33 | 0,55 | -7,55 | 8,96 |
| 36 | X |  |  |  | X | X |  |  | X |  | X | 19 | 7 | 43,50 | 32,58 | 53,91 | -1,52 | -10,50 | 6,91 |
| 37 |  |  | X |  | X | X |  |  | X |  | X | 189 | 84 | 43,70 | 37,07 | 50,37 | 0,36 | -6,33 | 8,07 |
| 38 |  |  | X | X |  |  | X |  | X | X |  | 35 | 16 | 44,20 | 34,23 | 54,21 | 0,80 | -7,48 | 9,44 |
| 39 |  | X |  |  | X |  | X |  | X |  | X | 23 | 6 | 44,71 | 32,01 | 55,23 | -3,31 | -13,72 | 4,81 |
| 40 | X |  |  |  | X |  | X |  | X | X |  | 91 | 47 | 45,39 | 37,12 | 54,12 | 2,38 | -5,24 | 11,53 |
| 41 | X |  |  | X |  | X |  |  | X |  | X | 10 | 4 | 46,23 | 34,77 | 57,56 | -0,86 | -10,39 | 8,33 |
| 42 |  |  | X | X |  | X |  |  | X |  | X | 204 | 101 | 47,11 | 40,67 | 53,70 | 1,71 | -4,43 | 8,83 |
| 43 |  | X |  | X |  |  | X |  | X |  | X | 31 | 12 | 47,82 | 37,11 | 57,92 | -2,28 | -11,41 | 5,85 |
| 44 | X |  |  | X |  |  | X |  | X | X |  | 142 | 83 | 51,42 | 43,10 | 60,15 | 6,37 | -1,29 | 15,40 |
| 45 | X |  |  |  | X |  | X |  | X |  | X | 29 | 15 | 52,98 | 41,94 | 64,19 | 0,09 | -8,84 | 9,71 |
| 46 |  |  | X | X |  |  | X |  | X |  | X | 50 | 26 | 53,22 | 43,75 | 62,66 | -0,06 | -8,31 | 8,06 |
| 47 | X |  |  | X |  |  | X |  | X |  | X | 25 | 14 | 55,18 | 44,23 | 65,74 | 0,23 | -8,70 | 8,94 |
| 48 |  |  | X |  | X |  | X |  | X |  | X | 149 | 95 | 57,46 | 48,82 | 66,45 | 6,27 | -0,87 | 16,02 |
| fem.: female, marr.: married, not marr.: not married, *: moderate or bad, CI: credible interval | | | | | | | | | | | | | | | | | | | |
