# Supplementary material for: Non-response in a national health survey in Germany: An intersectionality-informed multilevel analysis of individual heterogeneity and discriminatory accuracy
Source: PLoS One. 2020 Aug 10;15(8):e0237349. doi: 10.1371/journal.pone.0237349 (PMC7416954; doi:10.1371/journal.pone.0237349)
Supplement: S1 Appendix — (DOCX) [file pone.0237349.s003.docx]

**S1 Appendix: Comparison of the cross-sectional sample of DEGS1 with census data.**

|  | responder DEGS1 | | | | participants of non-responder survey of DEGS1 | | | | responder +  participants of non-responder survey of DEGS1 | | | | census data* | | |
| --- | --- | --- | --- | --- | --- | --- | --- | --- | --- | --- | --- | --- | --- | --- | --- |
|  | N | | % | | N | | % | | N | % | | | N^1^ | | % |
| Sex/gender |  | |  | |  | |  | |  |  | | |  | |  |
| male | 2016 | | 48.1 | | 1162 | | 49.6 | | 3178 | 48.6 | | | 31850 | | 49.7 |
| female | 2176 | | 51.9 | | 1180 | | 50.4 | | 3356 | 51.4 | | | 32254 | | 50.3 |
|  |  | |  | |  | |  | |  |  | | |  | |  |
| Marital status |  | |  | |  | |  | |  |  | | |  | |  |
| married | 2363 | | 57.0 | | 1208 | | 51.7 | | 3571 | 55.1 | | | 33494 | | 52.2 |
| not married | 1779 | | 43.0 | | 1129 | | 48.3 | | 2908 | 44.9 | | | 30610 | | 47.8 |
|  |  | |  | |  | |  | |  |  | | |  | |  |
| Educational level |  | |  | |  | |  | |  |  | | |  | |  |
| high | 2969 | | 71.6 | | 1416 | | 60.9 | | 4385 | 67.7 | | | 36642 | | 60.3 |
| low | 1177 | | 28.4 | | 911 | | 39.1 | | 2088 | 32.3 | | | 24173 | | 39.7 |
|  |  |  | |  | |  | |  | | |  |  | |  | |

^1^ N for census data divided by 1000

*census data was obtained from the internet portal of the German Federal Statistical Office:

data on sex/gender:

census data is based on the census 2011, extrapolated to 31.12.2010 (from age 18 to 79)

(source: Table Nr. “12411-0008”, <https://www-genesis.destatis.de/genesis/online>, accessed 12.11.2019)

data on marital status:

census data is based on the census 2011, extrapolated to 31.12.2010 (from age 18 to 79)

(source: Table Nr. “12411-0008”, <https://www-genesis.destatis.de/genesis/online>, accessed 12.11.2019)

data on educational level:

census data is based on the census 2011 for the effective date of 9.5.2011 (from age 20 to 79)

(source: Table „Personen nach Alter (5er-Jahresgruppen) und Höchster Schulabschluss für Deutschland)“, <https://ergebnisse.zensus2011.de/#dynTable:>, accessed 12.11.2019)
