# Supplementary material for: Non-response in a national health survey in Germany: An intersectionality-informed multilevel analysis of individual heterogeneity and discriminatory accuracy
Source: PLoS One. 2020 Aug 10;15(8):e0237349. doi: 10.1371/journal.pone.0237349 (PMC7416954; doi:10.1371/journal.pone.0237349)
Supplement: Attachment — Submitted filename: 20200617_Response to Reviewers.docx [file pone.0237349.s004.docx]

**Rebuttal Letter**

| Reviewer #1: This is a worthy study applying an intersectional framework to investigate non-participation in population health surveys. The use of MAIHDA is innovative and clearly suitable. The authors not only focus on the differences between group averages, but they also consider the individual heterogeneity around those averages by interpreting the VPC as a measure of discriminatory accuracy. In my opinion the analytical and conceptual approach used by the authors represent a new and improved way of performing epidemiological analyses. However, I have several comments. |  |
| --- | --- |
| R1: In the abstracts, the authors conclude they analyses “do not support targeting specific intersectional strata to achieve more equal study participation”. I think this is right but incomplete. The authors provide a better formulation in page 24, lines 343-345 “Our results indicate that there is no justification to target specific intersectional strata in order to increase response, but that a combination of targeted and population-based measures might be appropriate to achieve more equal representation”.  This concept may appear vague of confuse but it is a relevant issue. It does not mean specific intersectional strata are irrelevant. From an individual/clinical perspective the risk of non-participation in an strata with high average risk may be high. However, a low VPC (low discriminatory accuracy) indicates that focusing ONLY on strata with a low average participation would miss many individuals prone to low participation but belonging to strata with a high average participation. That is, using the strata averages as criteria for intervention may give many false negatives (as well as false positive) if the VPC is low. I suggest, the conclusions in lines 343-34 should also be in the abstracts ( you may add “only”), That is “Our results indicate that there is no justification to target specific intersectional strata ONLY in order to increase response, but that a combination of targeted and population-based measures might be appropriate to achieve more equal representation”. | Thank you very much for spotting this inconsistency. We would agree that the conclusion in the manuscript is more accurate given the results of our study. We modified the conclusion in the abstract accordingly. |
| R2: I think study non-participation by using a survey is interesting but a bit peculiar, as they are non-participants among the non-participants. I would rather use register data to obtain demographic and socioeconomic variables.  In any case you must provide a flow diagram indicating very clearly how many individuals were excluded and the reasons for the exclusion. The reader needs to understand this process very well. | Thank you very much for this comment. We are fully aware of this limitation and discussed it in detail in lines 297-308 of the marked-up manuscript. As we were interested in analyzing subjective health together with social variables, we decided to use data from the non-responder survey for this analysis. Data on subjective health was not available from census data.  Thank you for the suggestion to include a flow diagram, which is probably very helpful for readers to understand the selection of the sample. We created a flow diagram (Fig 1). |
| R3: the use of “subjective health” may not fit well intersectionality theory and some sociologists may consider it unsuitable if you do not explain how subjective health condition social location in intrinsic combination with other axes of oppression (see your reference #24 for further explanations).  I would take some distance from feministic intersectionality “theory” and explain the study is inspired on the intersectionality approach concerning socioeconomic variables, but it also adopts a multicategorical/stratified design to provide a better understanding of the heterogeneity in non-participation.  I think the purpose when using MAIHDA is (i) to provide an improved mapping of how non-participation is distributed across the intersectional strata in the population. When doing so, (ii) the method provides reliability weighted strata specific risks and, thereby, can handle strata with small number of individuals. In addition, (iii) intersectional MAIHDA allows to detect strata specific interaction of effects. On top of that, (iv) MAIHDA provides measures of DA that can be used when interpreting the observed differences in non-participation risk across strata. | Thank you for this comment and for the hint to further references.  We revisited the manuscript and tried to emphasize that we want to put some distance between this analysis and intersectionality theory. We therefore called our approach an “intersectionality-informed analysis” that aimed to apply MAIHDA to estimate proportions of non-response and heterogeneity in non-response across intersectional strata. These corrections can be found throughout the manuscript.  We added an explanation why subjective health might fit in our intersectionality-informed approach. In lines 129-137 and 324-327, we discuss why subjective health might condition social location. As this aspect of the paper might be viewed as controversial, we chose to present results of two scenarios, one without and one with subjective health when conceptualizing the analysis. By doing so, we intended to highlight that scenario one contains indisputable markers of social location that were used similarly in other MAIHDA studies.  We extended the discussion of the advantages of MAIHDA in lines 350-354. |
| R3: Regrading the methodology and results I have not mayor comments.  I think you should provide figures with points estimations and confidence intervals rather that only tables.  Please, provide % (n/N) rather than only % In your sample the problem of strata with very small number of individuals is not actual. However, you should indicate that an advantage of the multilevel analysis is that it uses shrunken residuals so small strata are handle much better than in traditional fixed effects regression. | Thank you for this comment. Since tables were very rich of information, we generated figures to present the results in a more accessible fashion (Fig 2-5). The old tables are included as supplements.  % (n/N) was provided in the results section where appropriate.  The important advantage of MAIHDA to handle strata with small sample size is mentioned in the discussion in lines 343-354. |
| R4: Finally, the cross-classified MAIHDA allow to separate geographical from socioeconomic influences and evaluate their relative relevance. This is interesting information and you should further discuss this analysis | We did not intend to separate geographical from socioeconomic influences in this paper. However, this is a very good point and we included this suggestion in the discussion in lines 337-342. In addition, we emphasized in the methods section that we used a cross-classified design to account for the clustered sampling design of DEGS1. |
